# Corpus luteum number and the maternal renin-angiotensin-aldosterone system as determinants of utero-placental (vascular) development: the Rotterdam Periconceptional Cohort

**DOI:** 10.1186/s12958-021-00843-9

**Published:** 2021-11-04

**Authors:** Rosalieke E. Wiegel, Maud J. H. Karsten, Igna F. Reijnders, Lenie van Rossem, Sten P. Willemsen, Annemarie G. M. G. J. Mulders, Anton H. J. Koning, Eric A. P. Steegers, A. H. Jan Danser, Régine P. M. Steegers-Theunissen

**Affiliations:** 1 Department of Obstetrics and Gynecology, Erasmus MC, University Medical Center, Dr. Molewaterplein 40, 3015 GD Rotterdam, The Netherlands; 2 Department of Biostatistics, Erasmus MC, University Medical Center, Rotterdam, The Netherlands; 3 Department of Pathology, Clinical Bioinformatics Unit, Erasmus MC, University Medical Center, Rotterdam, The Netherlands; 4 Department of Internal Medicine, Erasmus MC, University Medical Center, Rotterdam, The Netherlands

**Keywords:** Renin-angiotensin-aldosterone system, Prorenin, In-vitro fertilization, Corpus luteum, Hemodynamic adaptation, Placentation, First-trimester, Ultrasound, Virtual reality

## Abstract

**Background:**

Pregnancies with > 1 corpus luteum (CL) display a hyperdynamic circulation and an increased risk of small-for-gestational age deliveries. Among the factors released by the CL is prorenin, the inactive precursor of renin. Since the renin-angiotensin-aldosterone system (RAAS) is involved in early hemodynamic pregnancy adaptation, we linked both CL number and first-trimester concentrations of prorenin (as an indicator of RAAS activity) and the aldosterone/renin ratio (as an indicator of angiotensin-independent aldosterone effectiveness) to non-invasive markers of utero-placental (vascular) development, measured longitudinally from the first trimester onwards.

**Methods:**

A total of 201 women, who conceived naturally or after in-vitro fertilization treatment (with 0 (*n* = 8), 1 (*n* = 143), or > 1 (*n* = 51) CL), were selected from the Rotterdam Periconceptional Cohort. Maternal RAAS components were determined at 11 weeks gestation. Placental volume and utero-placental vascular volume were measured from transvaginal 3D ultrasound scans at 7, 9 and 11 weeks gestation, pulsatility and resistance indices of the uterine arteries were assessed by pulsed wave Doppler ultrasounds at 7, 9, 11, 13, 22 and 32 weeks gestation. At birth placental weight was obtained using standardized procedures.

**Results:**

Pregnancies without a CL show lower uterine artery indices throughout gestation than 1 CL and > 1 CL pregnancies, while parameters of placental development are comparable among the CL groups. After adjustment for patient- and treatment-related factors, first-trimester prorenin concentrations are positively associated with uterine artery pulsatility and resistance indices (β 0.06, 95% CI 0.01;0.12, *p* = 0.04 and β 0.10, 95% CI 0.01;0.20, *p* = 0.04, respectively), while high prorenin concentrations are negatively associated with first-trimester utero-placental vascular volume (β -0.23, 95% CI -0.44;-0.02, *p* = 0.04) and placental weight (β -93.8, 95%CI -160.3;-27.4, *p* = 0.006). In contrast, the aldosterone/renin ratio is positively associated with first-trimester placental volume (β 0.12, 95% CI 0.01;0.24, *p* = 0.04).

**Conclusions:**

The absence of a CL, resulting in low prorenin concentrations, associates with low uterine artery pulsatility and resistance, while high prorenin concentrations associate with a low utero-placental vascular volume and weight. These data support a scenario in which excess prorenin, by upregulating angiotensin II, increases uterine resistance, thereby preventing normal placental (vascular) development, and increasing the risk of small-for-gestational age deliveries. Simultaneously, high aldosterone concentrations, by ensuring volume expansion, exert the opposite.

**Supplementary Information:**

The online version contains supplementary material available at 10.1186/s12958-021-00843-9.

## Introduction

Preeclampsia and fetal growth restriction are severe placenta-related complications which affect approximately 8–10% of all pregnancies [1]. These complications not only impact fetal growth and pregnancy outcome, but also have consequences for long-term maternal and offspring health [2, 3]. The origin of inadequate fetal growth and development is often attributable to placental dysfunction [4, 5]. Placentation involves the development of the placental bed, which starts in the first trimester of pregnancy [6]. This time period requires trophoblast growth, invasion in the endometrium of the uterine wall, and remodeling of the uterine spiral arterioles to effectuate an adequate maternal blood flow to the placenta [7, 8].

Yet, a healthy pregnancy also relies on systemic alterations in the maternal cardiovascular and renal system, resulting in a hyperdynamic circulatory state. These alterations start in the luteal phase of the menstrual cycle, and involve factors released by the corpus luteum (CL). Indeed, pregnancies after in-vitro fertilization (IVF) treatment with frozen embryo transfer (FET) in a programmed cycle, lacking a CL, display attenuated systemic and renal adaptations during the first trimester, increasing their risk of developing preeclampsia [9–11], while pregnancies after controlled ovarian stimulation followed by fresh embryo transfer (ET) in the presence of multiple CL are at greater risk for small-for-gestational age (SGA) deliveries [12, 13]. It has already been suggested that this reflects the detrimental consequences of both a hypodynamic (0 CL) and hyperdynamic (> 1 CL) circulation, related to the release of too little or too much CL-derived factors into the circulation [9].

**Fig. 1:**
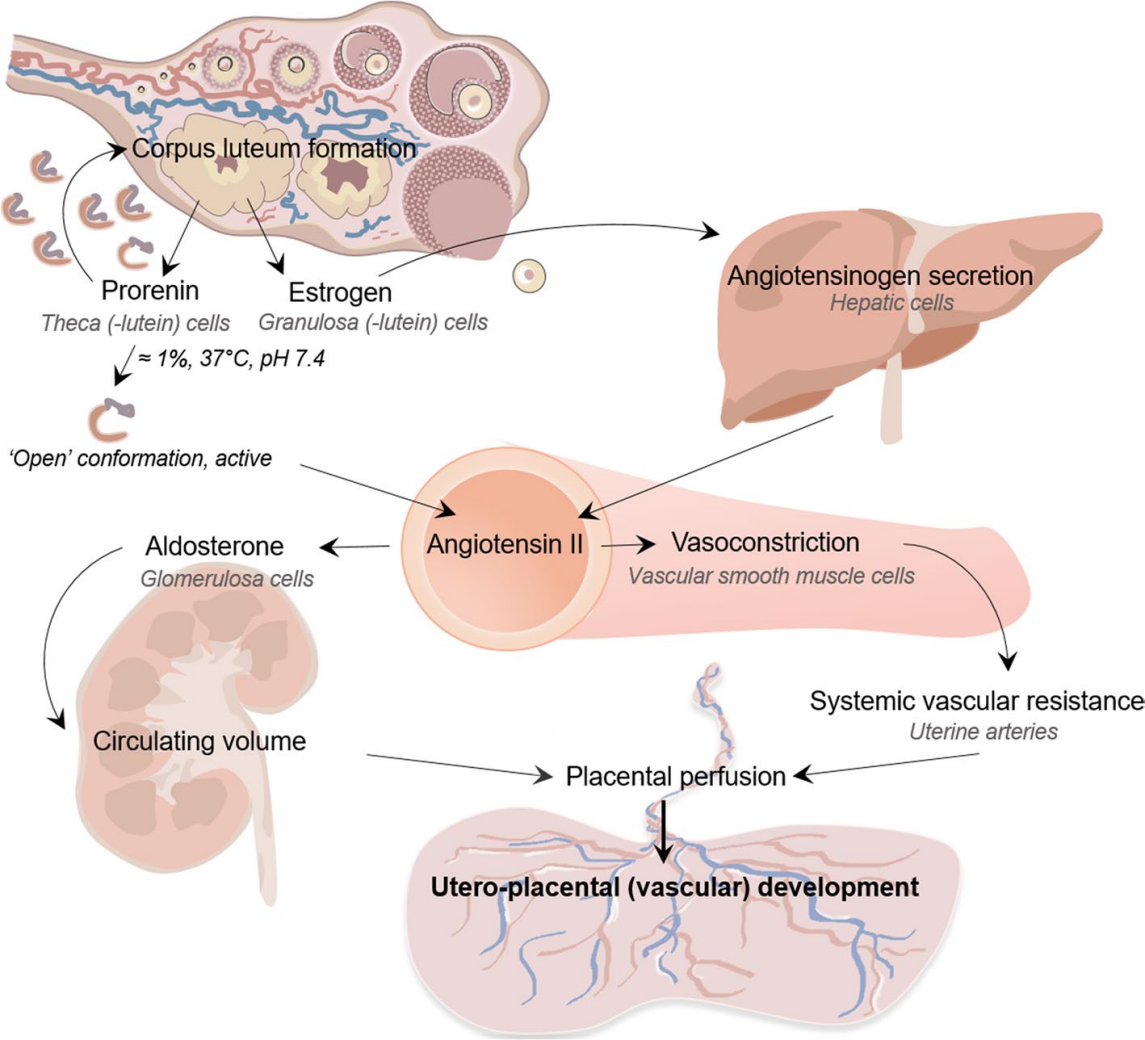
The impact of corpus luteum number, through excessive or limited maternal RAAS activation during early gestation, on early hemodynamic and cardiovascular adaption, affecting utero-placental (vascular) development throughout pregnancy. Under physiological conditions (pH 7.4, 37 °C), a small fraction of prorenin (≈1% or less) occurs in the so-called ‘open’ conformation, i.e., a conformation where the prosegment has moved out of the enzymatic cleft, thus allowing it to react with angiotensinogen to yield angiotensin I. Yet, the majority of prorenin is closed and inactive. Cleavage of the prosegment (also known as ‘proteolytic’ activation) occurs exclusively in the kidney, and depends on an as yet unidentified enzyme [16]. Abbreviations: RAAS = renin-angiotensin-aldosterone system

Among the factors secreted by the CL is prorenin, the inactive precursor of renin [14, 15]. Prorenin and renin concentrations increase with higher CL numbers, as shown by our previous research [14]. Why the CL secretes large amounts of prorenin remains unknown. Prorenin’s prosegment covers the active site, thus preventing it from displaying activity. Prosegment cleavage only occurs in the kidney. However, under physiological conditions (pH 7.4, 37 °C), a small percentage of prorenin (≈1%) is present in the so-called “open-conformation”, i.e., the prosegment has temporarily moved out of the enzymatic cleft (Fig. 1) [17]. Under such conditions, prorenin can react with angiotensinogen and generate angiotensin (Ang) I. A further possibility is that binding to a receptor facilitates this conformational change, allowing prorenin to display activity in tissues. Once Ang I is generated, it is converted to Ang II by angiotensin-converting enzyme. Ang II is not only an important blood pressure regulator with major effects on fluid and electrolyte balance, but it also stimulates the synthesis and release of aldosterone [18]. The latter is indispensable to achieve the 30–40% increase in plasma volume during pregnancy.

In the present study we speculated that renin-angiotensin-aldosterone system (RAAS) activation in the first trimester influences placental growth. We therefore linked first-trimester maternal RAAS component concentrations in pregnancies with varying CL numbers (0, 1 or > 1) to markers of utero-placental (vascular) growth and development that were measured longitudinally in a non-invasive manner from the first trimester onwards.

## Methods

### Study design

Participants were part of the ongoing prospective Rotterdam Periconceptional Cohort Study (Predict Study), performed at the Department of Obstetrics and Gynaecology of the Erasmus Medical Centre Rotterdam [19]. Between January 2017 and March 2018, this study enrolled a subcohort of 241 women for the VIRTUAL Placenta study (registration number Dutch Trial Register: NTR6854), which focuses on the (patho)physiology of early placental development by using non-invasive measurements for placental development during the first trimester. Women with an ongoing singleton pregnancy of less than 10^+^^0^ weeks of gestation, at least 18 years of age and familiar with the Dutch language, were eligible for participation. Pregnancies conceived either spontaneously (this also included conceptions after ovulation induction or intra-uterine insemination), or through IVF, or through intracytoplasmic sperm injection (ICSI), were eligible for inclusion. Participants were excluded in case of miscarriage, fetuses with congenital malformations, oocyte donation, and when no first-trimester blood samples were available. The VIRTUAL Placenta study has been approved by the Medical Ethical Committee of the Erasmus Medical Centre, and was performed conform the ethical principles of the Declaration of Helsinki. Participants signed informed consent before enrolment.

### Study parameters

After enrolment participants filled out a questionnaire which provided details about general characteristics like age, education, geographical origin, parity, pre-pregnancy weight and length to calculate the body mass index (BMI), medication, lifestyle (nutrition and use of cigarettes, alcohol and folic acid and/or multivitamins), and previous diagnosis of pregnancy complications. Use of cigarettes or alcohol was defined as any consumption during the periconception period, defined as 14 weeks prior to conception to 10 weeks after [20]. All data were verified at study entry by a research nurse and checked with medical records. Clinical assessments were performed at 7, 9, 11, 13, 22 and 32 weeks gestational age (GA). During each visit women underwent 3D power Doppler ultrasound scans of the placenta, pulsed-wave Doppler measurements of the right and left uterine artery, and weight and height were determined. Systolic and diastolic blood pressure were measured in a sitting position, on the right arm, by using a manual blood pressure cuff and stethoscope. Mean arterial pressure (MAP) in mmHg was calculated from the systolic blood pressure + 2*diastolic blood pressure, divided by 3. Details regarding subfertility diagnoses, type of fertility treatment and origin of gametes, were retrieved from medical records. Birth outcomes were obtained through the postpartum filled out questionnaires, which were cross-checked with medical records. Placenta-related complications were defined as pregnancy induced hypertension (PIH), preeclampsia (PE), preterm birth (PTB), fetal growth restriction (FGR) and/or SGA (<p10). The diagnoses of placenta-related complications were established by clinical symptoms and confirmed by pathologists. Hypertensive disorders were defined according to the recommendations of the American College of Obstetricians and Gynecologists (ACOG) [21]. Fetal growth restriction was defined as fetal abdominal circumference and/or estimated fetal weight below the 10th percentile according to Hadlock curves, or a more than twenty percentile decrease on the growth curve, compared to previous measurements with at least a gap of two weeks [22]. SGA was defined as birthweight below the 10th percentile on growth curves specific for GA at birth, parity and fetal sex and PTB as < 37 weeks GA [23]. Placental weight at birth was obtained according to standardized protocols.

### Assisted-reproduction protocols

Standard protocols were used for controlled ovarian stimulation, oocyte retrieval, IVF treatment, and assessment of embryo morphology [24, 25]. Pregnancies conceiving by ovarian stimulation were treated by either a gonadotropin-releasing hormone (GnRH) agonist or -antagonist followed by either Menopur, a highly purified, urine-derived, human menopausal gonadotropin (hMG, containing follicle-stimulating hormone [FSH], luteinizing hormone [LH] and human chorionic gonadotropin [hCG]) or recombinant human (rh)FSH (Bemfola, Gonal-F or Rekovelle). To induce final oocyte maturation and ovulation a single dose of hCG or a GnRH agonist was administered. Oocyte retrieval was performed 36 h after the hCG injection. Modified natural-cycle FETs were performed if patients had a regular menstrual cycle. A frozen-thawed ET was performed 4 days after spontaneous ovulation, detected by urinary LH testing. In case of absence or irregularity of the menstrual cycle, a programmed FET cycle was performed in a hormonally-prepared endometrium, by using estrogens followed by increasing dosages of intravaginal progesterone and optionally daily injections of a GnRH-agonist. Programmed FET cycles were monitored by ultrasound until the endometrium reached a size of 4 mm or larger. Frozen-thawed ET occurred after approximately 19 days of endometrial preparation.

In pregnancies conceived by IVF/ICSI, GA was determined on the day of transfer. GA was calculated based on the date of oocyte retrieval plus 14 days for pregnancies after fresh ET. In pregnancies after FET, GA was defined as the transfer date plus 19 days. Spontaneous pregnancies in this cohort occurred in women with and without known subfertility. In pregnancies after natural conception, GA was calculated based on the first day of the last menstrual period (LMP) in regular cycles between > 25 and < 32 days. In case of an irregular menstrual cycle, unknown LMP, or when GA based on LMP deviated more than six days from the GA estimated by on crown-rump-length, the GA was estimated using crown-rump-length.

### Biochemical measurements

Non-fasting venous blood samples (±750 μL) were obtained at each study visit and collected in vacutainer ethylenediaminetetraacetic acid (EDTA) tubes, processed, and stored at − 80 °C until analysis. Blood samples obtained at two times during the first trimester (median 9^+2^ weeks of gestation [IQR: 9^+1^–9^+4^] and median 11^+2^ weeks of gestation [IQR: 11^+1^–11^+5^]) were analysed at the Erasmus MC. Renin was measured by the immunoradiometric assay (Cisbio: detection limit 1 pg/mL; intra- and interassay coefficients 1.4 and 3.7%, respectively) using an active site-directed radiolabeled antibody that recognizes renin only. Total renin was measured with the same assay, after activating prorenin with 10 μmol/L aliskiren at 4 °C for 48 h. The amount of prorenin was calculated by subtracting renin from total renin. Aldosterone was measured by solid-phase radioimmunoassay (Demeditec Diagnostics: detection limit 11 pg/mL; intra- and interassay coefficients 2 and 5%, respectively).

### Ultrasound measurements and outcomes

**Fig. 2:**
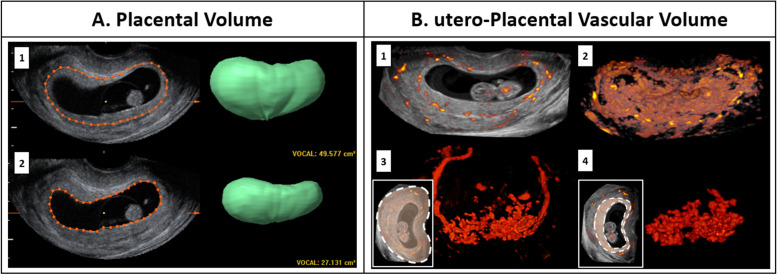
**A**: Measurement of placental volume (PV, cm^3^) using 3D ultrasound volumes and VOCAL measuring technique by subtracting 1. total pregnancy volume and 2. gestational sac volume. **B**: utero-placental vascular volume obtained by semi-automated measurements using three-dimensional power Doppler ultrasound images and Virtual Reality (VR) visualization. 1. Slice view of a complete 3D power Doppler vascular volume at 9 weeks GA, with surrounding grey values representing the uterine tissue. 2. Volume view of Fig. B1 showing the total obtained pregnancy volume, after setting a threshold for grey values. 3. Volume view of the complete 3D Power Doppler vascular volume. The anatomic orientation of the vasculature in color is demonstrated in the lower insert, delineated by a dashed white line. 4. Volume view of the utero-placental vascular volume (uPVV, cm^3^), calculated by subtracting uterine and fetal vascular volumes using a virtual brush to erase vascular voxels up to the myometrial-placental border, leaving the utero-placental vascular volume, delineated by the dashed with line

At 7, 9 and 11 weeks of gestation, participants underwent a transvaginal 3D Power Doppler ultrasound scan of the vascular bed from the complete gestational sac including the placenta, performed with a 6–12 MHz transvaginal probe compatible with the GE Voluson E8 and E10 Expert System (GE Medical Systems, Zipf, Austria). Placental volume (PV, cm^3^) was measured offline by using Virtual Organ Computer-aided AnaLysis (VOCAL™) (4D View, GE Medical System, Zipf, Austria) [26]. The VOCAL algorithm is first used to create a 3D volumetric segmentation of the total pregnancy (consisting of the placenta and the gestational sac), by interpolating between 12 manually drawn contours of the placenta in 2D planes with a rotational interval of 15°. Subsequently, a segmentation of the gestational sac volume was obtained in a similar way and finally the PV was calculated by subtracting the gestational sac volume from the total pregnancy volume (Fig. 2A).

Utero-Placental Vascular Volume (uPVV, cm^3^), was measured offline by making use of the Virtual Reality (VR) desktop system, enabling us to project the volume as a hologram, allowing accurate rotation and enlarging for removing the embryonic structures. The VR desktop system uses the V-Scope software to render the “holograms” and allows segmentation and volumetric measurements. Difference in echogenicity between the myometrium and placenta was used to select only the vessels (based on their power Doppler signal) up to the utero-placental border, and thereby creating a segmentation of the uPVV (Fig. 2B). This technique has been extensively described by Reijnders and colleagues [4], and is a reliable reflection of the uPVV of the maternal side of the placental bed with intra-class correlation coefficients > 0.94 [4]. Standardized settings for the power Doppler ultrasound were used to obtain scans of the utero-placental vasculature (power Doppler gain ‘-8.0’, pulse repetition frequency ‘0.6 kHz’, wall motion filter ‘low 1’, quality ‘high’) [4, 27]. Furthermore, the following default instrument settings were used throughout the examinations: frequency, low; dynamic, set 5; balance, 180; smooth, 4/5; ensemble, 12; line density, 8; power Doppler map, 5; artifact suppression, on; power Doppler line filter, 1; quality, high. In VR and 4D view, the quality of the acquired 3D (power Doppler) ultrasound volumes at each time point was scored on the presence of artifacts, acoustic shadowing, volume completeness, placental position in relation to the transducer and overall quality [4]. When images could not be optimally visualized they were excluded for VOCAL and VR measurements [4].

The pulsatility index (PI) and resistance index (RI) of the uterine artery (UtA) were measured bilaterally in threefold at week 7, 9, 11 and 13 of gestation by using transvaginal pulsed wave Doppler ultrasound. The mean of these indices at each measurement moment was used for analysis. At week 22 and 32 of gestation, these indices were measured transabdominally by a single researcher with expertise in second- and third-trimester ultrasound imaging. Additionally, a 2D ultrasound was performed to measure parameters of the fetal circulation, umbilical artery (UmbA) PI and RI.

### Study groups

The study participants were grouped according to number of CL at natural or assisted conception: 0 CL, after FET in a programmed cycle, 1 CL in spontaneous conceptions, including ovulation induction or intrauterine insemination or FET in a modified natural ovulatory cycle, and > 1 CL in ovarian stimulation with fresh ET. The CL status at conception in pregnancies conceived by ovarian stimulation and fresh ET is based on the number of retrieved oocytes during ovum pick-up (median: 11 [IQR: 7–16]).

### Statistical analysis

Maternal demographic and clinical characteristics and pregnancy outcomes of the total study population, stratified for CL number, where analyzed by using mean ± SD for normal distributed variables, median and interquartile range for skewed variables and frequencies (proportions) for categorical variables. To test for difference, we used the Student’s t-test, Kruskal-Wallis test or the Chi-squared test, respectively. To obtain a normal distribution, maternal RAAS determinants and non-volumetric outcome parameters (UtA-RI, UtA-PI, MAP, UmbA-RI and UmbA-PI) were transformed to the natural logarithmic scale and third root transformation was applied for volumetric parameters (PV and uPVV) to establish linearity between our parameters and GA. Continuous data at each time visit for each parameter (PV, uPVV, UtA-RI, UtA-PI, MAP, UmbA-RI and UmbA-PI) were summarized by mean and standard deviation, and compared among the CL groups by Student’s t-test. Subgroup analysis focused on comparison of those parameters for only FET pregnancies, programmed cycle (0 CL) versus modified natural cycle (1 CL).

Our second aim focused on studying the association between RAAS component concentrations and longitudinal measurements of first-trimester PV and uPVV (7, 9 and 11 weeks GA) and additionally second- and third trimester UtA-RI, UtA-PI and MAP (7, 9, 11, 13, 22 and 32 weeks GA), by using linear mixed models. To extend analyses with the maternal circulation into the fetal circulation, linear mixed models were used to examine the association between RAAS components and trajectories of second- and third trimester UmbA indices (22 and 32 weeks GA). To minimize confounding by GA, only strictly dated pregnancies (*n* = 151), dated based on the LMP or date of ET, were included in the analyses with first-trimester placental parameters (PV and uPVV). Finally, linear regression was applied to examine the association between RAAS determinants and placental weight at birth. First, the models were only adjusted for gestational age (Model 1), either at time of the ultrasound or at time of delivery. Subsequently, the model was adjusted for important patient and fertility treatment characteristics, such as CL number, maternal age, BMI, parity, smoking status, fetal gender and the diagnosis of polycystic ovary syndrome (Model 2). Relevant confounders were defined by literature search, and a correlation matrix was used to decide which confounders would be applied to analyses. Finally, to estimate the impact of pregnancies complicated by placenta-related complications on the associations, we additionally adjusted Model 2 for these complications. Since all results were identical after this adjustment, we do not report these additional analyses.

Concentrations of RAAS generally increase throughout pregnancy, which is most likely caused by the natriuretic effects of progesterone and the increased glomerular filtration rate [18]. In our study, participants were included and blood was taken twice at 9 and 11 weeks of gestation. Given the fact that all RAAS component concentrations at 9 weeks were significantly correlated with their concomitant concentrations measured at 11 weeks gestation, we decided to perform the analyses with the largest number of available measurements, present at 11 weeks. Blood withdrawal around 11 weeks occurred in a very narrow time range (11^+ 3^ [IQR: 11^+ 2^–11^+ 6^), and thus no adjustment for time of blood draw was made.

Finally, given the fact that renin and prorenin are highly correlated, we chose to use the prorenin concentrations in our models to evaluate effects mediated via the Ang II pathway. Here, our consideration was that prorenin concentrations are higher than renin concentrations, and therefore less likely to be affected by the modest changes that might occur during storage, i.e., the so-called cold-induced prorenin ‘activation’, resulting in a small percentage of prorenin being recognized as renin [16]. Given that prorenin concentrations are > 10-fold higher than renin concentrations, such activation would affect renin more strongly than prorenin. Secondly, to evaluate effects mediated via aldosterone, independently of the Ang II pathway, we used the aldosterone/renin ratio. *P*-values < 0.05 were considered statistically significant. All analyses were performed in R (R for Windows, version 3.5; R Core Team).

## Results

### Baseline characteristics

The VIRTUAL Placenta study enrolled 241 pregnancies. After exclusion of 22 pregnancies ending in miscarriage, 4 pregnancies with oocyte donation, 8 with congenital malformations, 5 participants without first-trimester blood samples and one drop-out, 201 singleton ongoing pregnancies were selected for analysis. A total of 389 3D first-trimester ultrasound datasets were included, of which 283 (72.8%) were of sufficient quality for VOCAL measurements and 292 (75.1%) for VR measurements. Additionally, a total of 1092 UtA indices were obtained from 2D pulsed wave Doppler ultrasounds. Table 1 shows the maternal baseline characteristics and pregnancy outcomes of the total study population, stratified according to CL number at the moment of conception [14]. The mean maternal age was 32.2 years, 115 women were nulliparous and 82 conceived through IVF/ICSI treatment. Of the IVF/ICSI conceived pregnancies, 51 occurred after fresh ET (> 1 CL), and 23 and 8 by FET in a natural cycle (1 CL) or programmed cycle (0 CL), respectively. Of the 119 spontaneously conceived pregnancies (1 CL), 7 were achieved by intrauterine insemination and 8 by ovulation induction treatment. A total of 50 (24.9%) pregnancies developed placenta-related complications (9 PIH, 7 PE, 21 PTB, 14 FGR and/or 19 SGA). The 1 CL group displayed a higher maternal BMI, was less often nulliparous and less often used preconception folic acid supplements adequately than the 0 CL and > 1 CL groups. The CL groups were comparable with regard to maternal age, geographic origin, educational level and smoking. The maternal plasma concentrations for renin, prorenin and aldosterone were available at 9 and 11 weeks GA and have been reported before [14]. The RAAS concentrations differed significantly among the three CL groups (Table 1). As the concentrations at 9 (*n* = 111) and 11 weeks (*n* = 192) GA were strongly correlated and a larger number of measurements were available at 11 weeks, we continued all analyses with the RAAS concentrations at 11 weeks gestation.

**Table 1:** Baseline demographic and clinical maternal and fetal characteristics of the study population stratified by corpus luteum number

Characteristic	Total study population (*n* = 201)	0 CL (*n* = 8)	1 CL (*n* = 142)	> 1 CL (*n* = 51)	*p-value*
*Maternal*					
Age, years	32.2 ± 4.5	30.5 ± 3.9	32.0 ± 4.4	33.0 ± 4.7	0.25
Nulliparous (n, %)	115 (57.2)	6 (75.0)	68 (47.6)	42 (82.4)	< 0.01*^3^
Mode of conception, IVF/ICSI (n, %)	82 (40.8)				
Frozen ET, programmed cycle	8 (9.8)				
Frozen ET, natural cycle	23 (28.0)				
Fresh ET	51 (62.2)				
GA at first visit, days	55 [51, 65]	54 [51, 58]	62 [52, 65]	53 [50, 63]	0.021*^3^
Geographic origin (n, %)					0.24
Dutch	156 (79.2)	6 (75.0)	104 (75.4)	46 (90.2)	
Western other	6 (3.0)	0 (0.0)	5 (3.6)	1 (2.0)	
Non-western	35 (17.8)	2 (25.0)	29 (21.0)	4 (7.8)	
Educational level (n, %)					0.53
Low	18 (9.1)	0 (0.0)	14 (10.1)	4 (7.8)	
Middle	65 (33.0)	4 (50.0)	41 (29.7)	20 (39.2)	
High	114 (57.9)	4 (50.0)	83 (60.1)	27 (52.9)	
BMI at first visit in first trimester, measured (kg/m^2^)	24.9 [22.2;28.5]	21.4 [20.9;22.8]	25.2 [22.6;29.2]	23.9 [21.8;27.4]	0.02*^1^
MAP at first visit in first trimester, measured (mmHg)	80.3 ± 7.2	80.8 (12.1)	80.7 (6.8)	79.6 (7.1)	0.74
Folic acid supplement use, (n, %)	198 (98.5)	8 (100.0)	139 (97.9)	51 (100.0)	1.00
Preconception initiation (n, %)	165 (82.1)	8 (100.0)	110 (77.5)	51 (100.0)	0.001*^3^
Alcohol consumption (n, %)	57 (28.4)	5 (62.5)	42 (29.4)	10 (19.6)	0.04*^2^
Smoking (n, %)	27 (13.4)	1 (12.5)	21 (14.7)	5 (9.8)	0.68
Vitamin supplement use (n, %)	118 (58.7)	4 (50.0)	83 (58.5)	31 (60.8)	0.16
*First-trimester blood measurements*					
9 weeks GA, *n* = 111					
Renin, pg/mL	25 [18;33]	10 [9;14]	25 [18;32]	30 [23;38]	< 0.001*^1–3^
Prorenin, pg/mL	277 [201;380]	107 [75;134]	264 [191;365]	373 [270;443]	< 0.001*^1–3^
Aldosterone, pg/mL	357 [216;571]	323 [214;469]	297 [185;496]	533 [348;866]	< 0.001*^2,3^
11 weeks GA, *n* = 192					
Renin, pg/mL	25 [17;32]	12 [11;17]	23.4 [17;31]	27.0 [23;34]	0.001*^1–3^
Prorenin, pg/mL	266 [206;386]	129 [109;152]	245.2 [204;340]	351 [278;465]	< 0.001*^1–3^
Aldosterone, pg/mL	314 [222;465]	360 [266;411]	288 [209;398]	454 [301;727]	0.002*^3^
*Birth outcomes*					
GA at birth, days	39^+ 1^ [38^+ 0^;40^+ 0^]	39^+ 0^ [38^+ 6^;40^+ 0^]	39^+ 0^ [37^+ 6^;39^+ 5^]	39^+ 2^ [37^+ 3^;40^+ 4^]	0.19
Birthweight, gram	3330 [2975;3570]	3490 [3243; 3530]	3305 [2939; 3562]	3335 [3045; 3682]	0.70
Placental weight, gram	455 [387;569]	380 [260; 426]	438 [360; 514]	491 [433; 563]	0.34
Fetal sex, girls (n, %)	94 (47.7)	3 (42.9)	64 (46.0)	27 (52.9)	0.91
Placenta-related complication**(n, %)	50 (24.9)	1 (12.5)	39 (27.5)	10 (19.6)	0.38
PIH	9 (4.5)	0 (0.0)	7 (4.9)	2 (3.9)	0.79
PE	7 (3.5)	0 (0.0)	6 (4.2)	1 (2.0)	0.65
FGR	14 (7.0)	0 (0.0)	11 (7.7)	3 (5.9)	0.67
PTB (iatrogenic and spontaneous)	21 (10.4)	1 (12.5)	17 (11.9)	3 (5.9)	0.47
SGA	19 (9.5)	0 (0.0)	16 (11.2)	3 (5.9)	0.35

Table is partially based on previously published data [14]. Data are presented as mean ± SD or median (interquartile range). *Significance at *p* < 0.05. **Overlapping diagnoses. 1, statistical difference between 0 CL and 1 CL; 2, statistical difference between 0 CL and > 1 CL; 3, statistical difference between 1 CL and > 1 CL. CL = corpus luteum; GA = gestational age; ICSI = intracytoplasmic sperm injection; IVF = in vitro fertilization; ET = embryo transfer; BMI = body mass index; MAP = mean arterial pressure; PE = preeclampsia; PIH = pregnancy-induced hypertension; PTB = preterm birth; SD = standard deviation; SGA = small-for-gestational age, defined as birth weight below the 10th percentile. FGR = fetal growth restriction, defined as abdominal circumference and/or estimated fetal weight (EFW) below the 10th percentile or a more than twenty-percentile decrease on the growth curve, compared to previous measurements with at least a time span of two weeks

### Utero-placental (vascular) development parameters among the CL groups

**Fig. 3:**
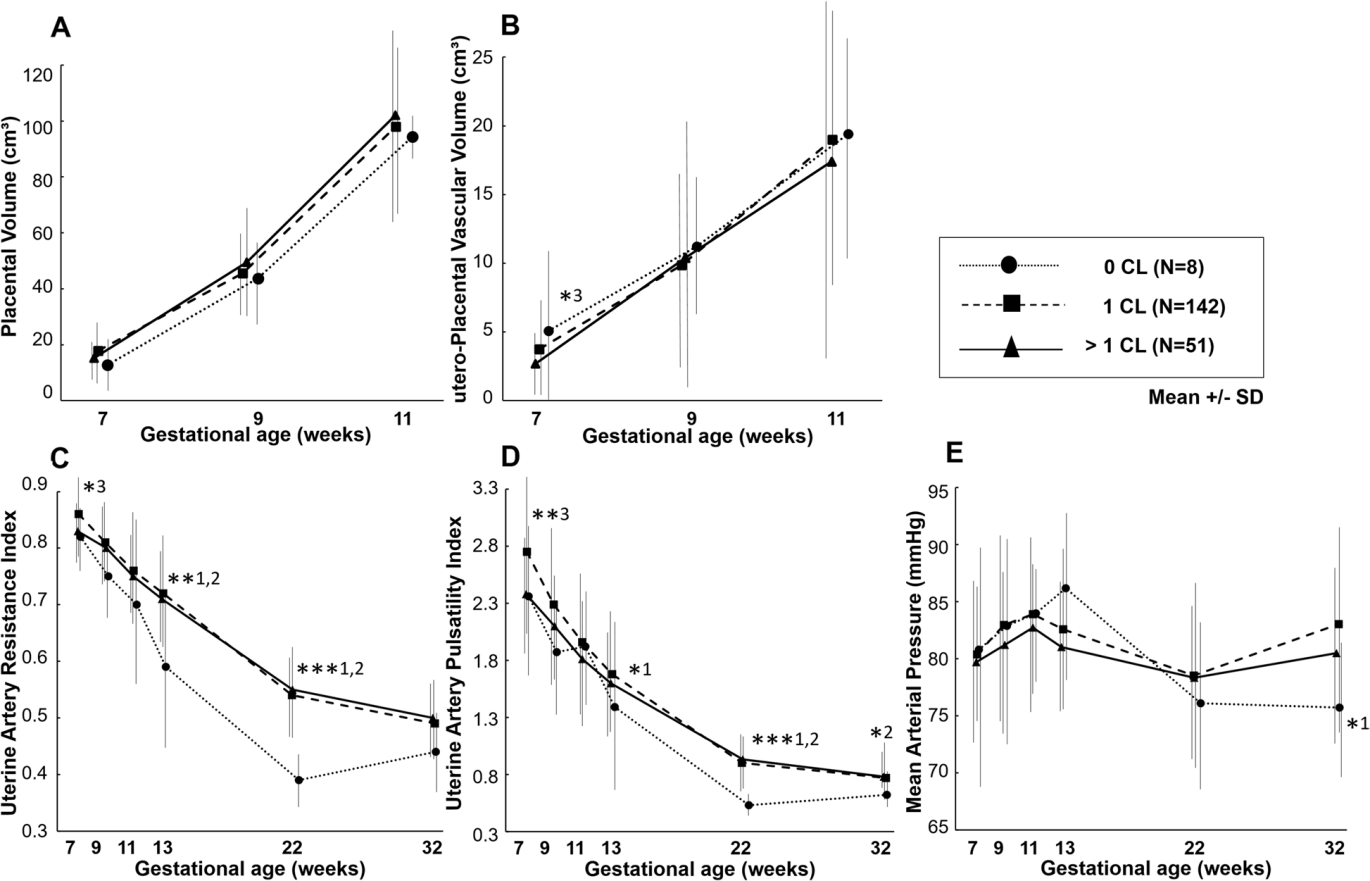
Changes in trajectories of placental volume (**a**), utero-placental vascular volume (**b**), uterine artery resistance (**c**) and pulsatility (**d**) indices and mean arterial pressure (**e**) during pregnancy in women with different number of corpora lutea. Mean ± SD. Student t-test was used to compare the CL cohorts at each time point separately. ***p < 0.001, **p < 0.01, *p < 0.05. 1, statistical difference between 0 CL and 1 CL; 2, statistical difference between 0 CL and > 1 CL; 3, statistical difference between 1 CL and > 1 CL. Abbreviations: CL = corpus luteum; SD = standard deviation

Differences in utero-placental (vascular) development parameters throughout gestation among the CL groups are presented in Fig. 3 and Table 2. The parameters PV and uPVV measured between weeks 7–11 did not differ between the 3 groups, although at 7 weeks uPVV in the > 1 CL group was lower compared to the 1 CL group. Pregnancies without a CL showed lower UtA-RI and PI throughout pregnancy than 1 CL and > 1 CL pregnancies, particularly in the second and third trimester of pregnancy. Although MAP was lower at 32 weeks gestation in the 0 CL group compared to the 1 CL group, no other differences between the 3 groups occurred. Results were similar if the 1 CL group was limited to the 23 FET pregnancies conceiving in a natural cycle with 1 CL (data not shown). Additionally, no CL-related differences in UmbA-RI and PI throughout the second and third trimester and placental weight at birth were observed.

**Table 2:** Longitudinal study of utero-placental (vascular) development trajectories and placental weight stratified by corpus luteum number

		7 weeks	9 weeks	11 weeks	13 weeks	22 weeks	32 weeks	At birth
**GA (days)**	0 CL	52 [51;54]	67 [65;67]	81 [79;81]	96 [94;97]	160 [157;161]	227 [226;230]	273 [271;280]
	1 CL	51 [50;54]	66 [65;67]	79 [78;82]	93 [92;94]	157 [155;159]	226 [224;228]	273 [265;279]
	> 1 CL	51 [50;54]	66 [65;67]	79 [78;82]	93 [92;94]	157 [155;159]	226 [224;228]	275 [269;284]
**PV (cm**^**3**^**)**	0 CL	12.8 ± 8.7	43.7 ± 15.1	94.4 ± 8.8				
	1 CL	17.8 ± 10.7	45.5 ± 16.4	98.0 ± 33.8				
	> 1 CL	15.2 ± 7.7	49.5 ± 21.8	102.1 ± 34.7				
**uPVV (cm**^**3**^**)**	0 CL	5.1 ± 7.3	11.2 ± 4.9	19.4 ± 8.6				
	1 CL	3.7 ± 3.3	9.9 ± 7.0	19.0 ± 10.5				
	> 1 CL	2.7 ± 2.7*^3^	10.4 ± 10.1	17.4 ± 14.2				
**UtA RI**	0 CL	0.82 ± 0.06	0.75 ± 0.07	0.70 ± 0.15	0.59 ± 0.14**^1,2^	0.39 ± 0.05***^1,2^	0.44 ± 0.08	
	1 CL	0.86 ± 0.08	0.81 ± 0.09	0.76 ± 0.10	0.72 ± 0.10	0.54 ± 0.09	0.49 ± 0.09	
	> 1 CL	0.83 ± 0.05*^3^	0.80 ± 0.07	0.75 ± 0.08	0.71 ± 0.09	0.55 ± 0.09	0.50 ± 0.07	
**UtA PI**	0 CL	2.36 ± 0.66	1.87 ± 0.53	1.92 ± 1.46	1.39 ± 0.85*^1^	0.53 ± 0.09***^1,2^	0.62 ± 0.16*^2^	
	1 CL	2.75 ± 0.78	2.29 ± 0.73	1.96 ± 0.69	1.68 ± 0.60	0.90 ± 0.30	0.77 ± 0.27	
	> 1 CL	2.38 ± 0.50**^3^	2.10 ± 0.46	1.81 ± 0.60	1.60 ± 0.52	0.93 ± 0.29	0.78 ± 0.21	
**MAP (mmHg)**	0 CL	80.8 ± 12.1	82.9 ± 9.9	84.0 ± 5.1	86.2 ± 8.7	76.1 ± 8.2	75.7 ± 6.7*^1^	
	1 CL	80.4 ± 7.0	83.0 ± 8.1	83.9 ± 8.1	82.6 ± 7.5	78.5 ± 8.7	83.0 ± 9.3	
	> 1 CL	79.7 ± 7.2	81.2 ± 7.1	82.7 ± 7.1	81.0 ± 6.5	78.3 ± 6.7	80.5 ± 8.1	
**UmbA RI**	0 CL					0.68 ± 0.04	0.61 ± 0.05	
	1 CL					0.70 ± 0.05	0.61 ± 0.07	
	> 1 CL					0.68 ± 0.06	0.63 ± 0.07	
**UmbA PI**	0 CL					1.09 ± 0.13	0.91 ± 0.11	
	1 CL					1.15 ± 0.16	0.94 ± 0.16	
	> 1 CL					1.11 ± 0.17	0.98 ± 0.16	
**Placental Weight (gram)**	0 CL							380 [260;426]
	1 CL							438 [360;514]
	> 1 CL							491 [433;563]

Data are presented as (mean ± SD) or median (interquartile range). ***p < 0.001, **p < 0.01, *p < 0.05. 1, statistical difference between 0 CL and 1 CL; 2, statistical difference between 0 CL and > 1 CL; 3, statistical difference between 1 CL and > 1 CL. CL = corpus luteum; GA = gestational age; PV = placental volume; uPVV = utero-placental vascular volume; UtA = uterine artery; RI = resistance index; PI = pulsatility index; MAP = mean arterial pressure; UmbA = umbilical artery

### Maternal first-trimester RAAS activation and utero-placental (vascular) development

**Fig. 4:**
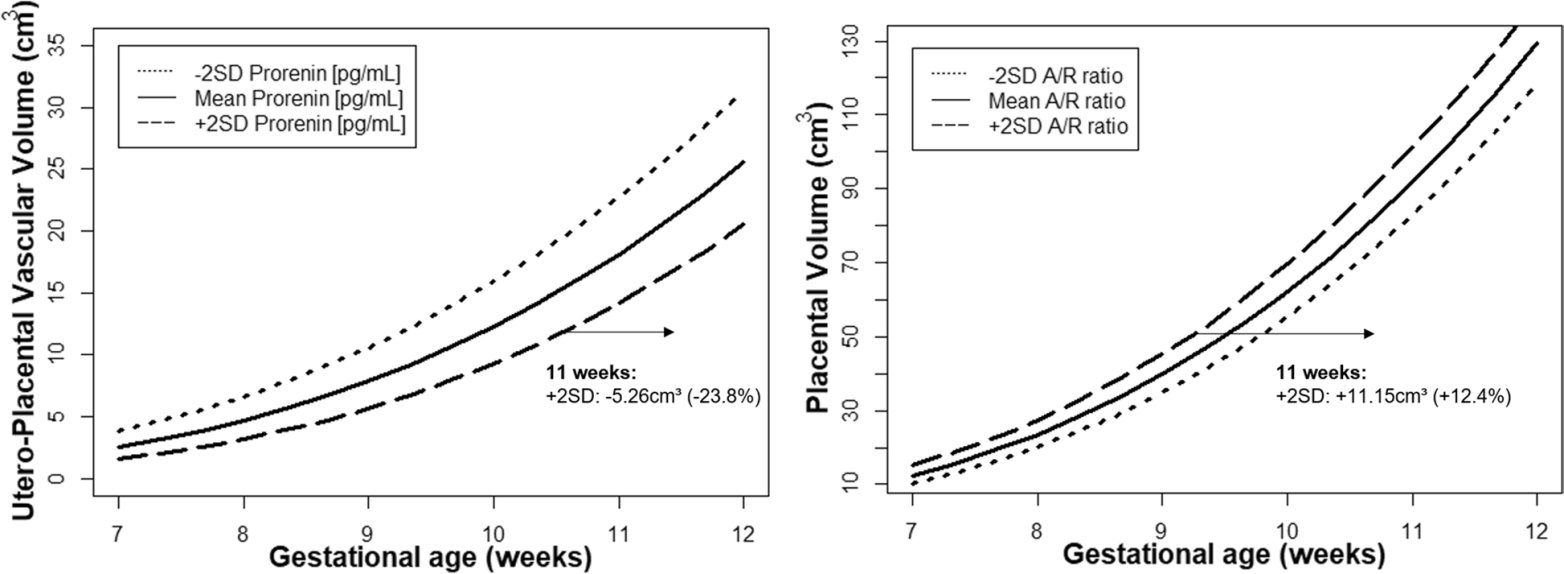
Utero-Placental (vascular) Volume trajectories for pregnancies with higher (mean + SD) and lower (mean – SD) prorenin concentrations or aldosterone/renin ratio at baseline for 11 weeks gestation in the total study population. In the fully adjusted model 2, high maternal prorenin concentrations (mean + 2SD) corresponds to a lower utero-placental vascular volume (at 11 weeks gestation -5.26 cm^3^) and a high A/R ratio (mean + 2SD) corresponds to a larger placental volume (at 11 weeks gestation +11.15 cm^3^) compared to the mean A/R ratio. Full adjustment includes gestational age, corpus luteum number, maternal age, parity, BMI, smoking, fetal gender and PCOS. Abbreviations: SD = standard deviation. A/R ratio = aldosterone/renin ratio

Table 3 shows the associations between the RAAS determinants and longitudinal utero-placental (vascular) development. Prorenin associated negatively with the first-trimester trajectory of uPVV in Model 2 (i.e., adjusted for gestational age, CL number, maternal age, BMI, parity, smoking status, fetal gender and the diagnosis of polycystic ovary syndrome), while the aldosterone/renin ratio associated positively with the first-trimester trajectory of PV in both Model 1 (adjusted for gestational age only) and Model 2. Retransformation of the effect estimates to the original scales shows that uPVV for pregnancies with higher maternal prorenin (mean + 2SD) concentrations was diminished by 5.26 cm^3^ (− 23.8%) at 11 weeks of gestation compared to the women with mean concentrations of prorenin. PV for pregnancies displaying a higher aldosterone/renin ratio (mean + 2SD) was increased with 11.15 cm^3^ (+ 12.4%) at 11 weeks of gestation compared to the women with the mean aldosterone/renin ratio (Fig. 4).

**Table 3:** Associations between first-trimester maternal RAAS concentrations and trajectories of utero-placental (vascular) development and placental weight

		Model 1		Model 2	
		Beta (95% CI)	*p*-value	Beta (95% CI)	*p*-value
**First-trimester trajectories** (*n* = 151)					
**PV (∛cm**^**3**^**)**	Log [Prorenin (pg/mL)]	-0.04 (-0.19; 0.12)	0.65	-0.14 (-0.33; 0.06)	0.16
	Log [Aldosterone/renin ratio]	0.12 (0.01; 0.22)	0.04*	0.12 (0.01; 0.24)	0.04*
**uPVV (∛cm**^**3**^**)**	Log [Prorenin (pg/mL)]	-0.08 (-0.27; 0.10)	0.38	-0.23 (-0.44; -0.02)	0.04*
	Log [Aldosterone/renin ratio]	0.003 (-0.13; 0.14)	0.96	0.01 (-0.13; 0.14)	0.91
**Total pregnancy trajectories** (*n* = 201)					
**UtA-RI**	Log [Prorenin (pg/mL)]	0.04 (0.001; 0.07)	0.04*	0.10 (0.01, 0.20)	0.04*
	Log [Aldosterone/renin ratio]	-0.02 (-0.05; 0.003)	0.08	-0.01 (-0.04; 0.01)	0.27
**UtA- PI**	Log [Prorenin (pg/mL)]	0.05 (-0.03, 0.12)	0.21	0.06 (0.01, 0.12)	0.04*
	Log [Aldosterone/renin ratio]	-0.05 (-0.10, 0.01)	0.08	-0.03 (-0.09, 0.02)	0.25
**MAP (mmHg)**	Log [Prorenin (pg/mL)]	-0.03 (-0.06, -0.01)	0.002*	-0.02 (-0.04, 0.01)	0.14
	Log [Aldosterone/renin ratio]	0.01 (-0.01, 0.02)	0.34	0.01 (-0.01, 0.03)	0.19
**Second-and third trimester trajectories** (*n* = 201)					
**UmbA-RI**	Log [Prorenin (pg/mL)]	0.01 (-0.004, 0.03)	0.15	0.02 (-0.001, 0.04)	0.06
	Log [Aldosterone/renin ratio]	-0.01 (-0.02, 0.01)	0.31	-0.004 (-0.02, 0.01)	0.55
**UmbA-PI**	Log [Prorenin (pg/mL)]	0.04 (-0.004, 0.08)	0.08	0.05 (0.01, 0.11)	0.04*
	Log [Aldosterone/renin ratio]	-0.02 (-0.05, 0.01)	0.24	-0.01 (-0.05, 0.02)	0.43
**At Birth** (*n* = 82)					
**Placental weight (gram)**	Log [Prorenin (pg/mL)]	-50.88 (-109.52; 7.76)	0.09	-93.82 (-160.30; -27.36)	0.006*
	Log [Aldosterone/renin ratio]	24.72 (-16,77; 66.21)	0.24	36.22 (-7.02; 79.45)	0.08

Table shows the effect estimates of the repeated measurements model for the associations of logarithmic transformed maternal total prorenin and aldosterone/renin ratio concentrations at 11 weeks gestation with trajectories of PV and uPVV during the first-trimester, UtA RI/PI and MAP throughout pregnancy and UmbA RI/PI during the second-and third trimester in the total study population and the linear model with placental weight at birth. Model 1: adjusted for gestational age, Model 2: adjusted model for gestational age, corpus luteum number, maternal age, maternal smoking, BMI, parity, fetal gender and polycystic ovary syndrome.*p < 0.05. CI = confidence interval; PV, placental volume; uPVV, utero-placental vascular volume; UtA, uterine artery; RI, restistance index; PI, pulsatility index; MAP, mean arterial pressure; UmbA = umbilical artery

First-trimester prorenin concentrations, like a higher CL number, associated positively with UtA-RI and PI throughout pregnancy (Table 3). No such associations were observed for the aldosterone/renin ratio. The negative prorenin-MAP relationship observed in Model 1 did not hold in Model 2. We stress here that when performing the analyses with total renin (i.e., renin + prorenin), results were the same (Supplemental Table 1; Supplemental Fig. 1).

### Maternal first-trimester RAAS activation and fetal circulation

A total of 376 UmbA indices were obtained from 2D pulsed wave Doppler ultrasounds during the second and third trimester. Prorenin (and total renin) associated positively with UmbA-RI and PI during the second and third trimester (Table 3; Supplemental Table 1). No associations with the aldosterone/renin ratio were observed.

### Placental weight

**Fig. 5:**
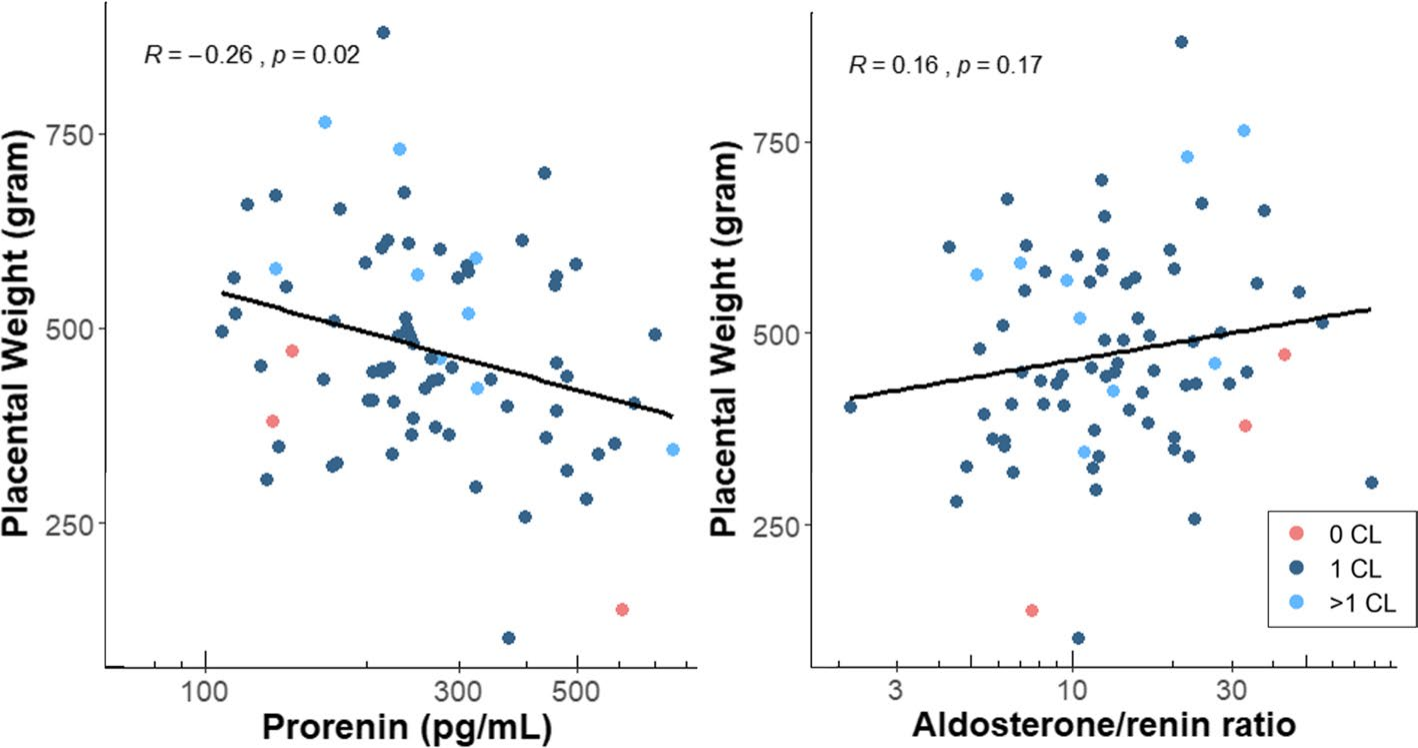
Correlations by Spearman Rank (*R*) test between maternal plasma prorenin concentrations and the aldosterone/renin ratio at 11 weeks gestation and placental weight at birth (*n* = 82) by different number of corpora lutea. Abbreviations: CL = corpus luteum

Placental weight after delivery was available for 82 participants, and the median placental weight was 455 [IQR: 387–569] gram. Prorenin associated negatively with placental weight in Model 2, while no association with the aldosterone/renin ratio was observed (Table 3; Supplemental Table 1). Finally, prorenin (and total renin) correlated negatively with placental weight (*R* = -0.26, *p* = 0.02), no significant correlation was observed with the aldosterone/renin ratio (Fig. 5; Supplemental Fig. 2).

## Discussion

This explorative study shows that both CL number and first-trimester prorenin concentrations associate positively with UtA-RI and PI throughout pregnancy. Prorenin, but not CL number, also associated positively with UmbA-RI and PI. Furthermore, high prorenin concentrations associated negatively with placenta weight and first-trimester uPVV, while aldosterone, when corrected for renin, associated positively with first-trimester PV. Potentially, these observations might underlie the relationship between a high CL number and the risk of developing placenta-related complications, in particular SGA. Conrad and Baker earlier proposed this to be linked to a hyperdynamic circulation [9], which could be the consequence of too much RAAS activation.

Physiologically, UtA-RI and PI decline progressively during pregnancy due to the decrease of vascular resistance in the placenta. The fact that these indices are lowest at low prorenin concentrations is suggestive for the prorenin-Ang II axis contributing to resistance (Fig. 1). This is logical given that Ang II is a potent vasoconstrictor. Since the CL is an important contributor to the prorenin concentrations in pregnancy, it is not surprising that the lowest UtA-RI and PI are seen in pregnancies with 0 CL. Normally, increased UtA indices increase the risk to develop preeclampsia or preeclampsia with severe features [28, 29]. However, an increased preeclampsia risk is also seen in women with 0 CL [13, 30, 31], who display low prorenin concentrations and low UtA indices. This supports the concept that a lack of CL results in the diminished presence of CL-derived factors (e.g., relaxin), which, like a high uterine artery resistance, contribute to the development of preeclampsia. In fact, it is well-known that RAAS activity is low in women with preeclampsia [32]. FET has been reported before to either result in a stronger or identical decline in UtA-PI versus fresh ET [33–35]. However, since these data were either not corrected for the endocrine uterine environment (and thus the CL number), or derived from IVF pregnancies obtained after oocyte donation, they cannot be directly compared with our results. Furthermore, although a lower BMI has been linked to a greater drop in UtA-PI [36], our results were independent of BMI (Supplemental Table 2), and thus the lower BMI in women with 0 CL cannot explain this outcome.

Normally, progesterone and prostacyclin make pregnant women refractory to Ang II [32], so that at least twice as much Ang II is needed to achieve the same hypertensive effect as in non-pregnant women [37]. Nevertheless, prorenin, if sufficiently elevated, might elevate Ang II up to levels that can no longer be counteracted sufficiently by either progesterone or prostacyclin. The resulting impaired perfusion of the utero-placental vascular bed is likely to underlie the higher risk of SGA and lower placental weight in pregnancies with multiple CL [38]. Indeed, animal studies confirm that Ang II infusion increases uterine and umbilical vascular resistance and reduces blood flow to the placenta and umbilical cord [39, 40]. Therefore it is not surprising that we additionally observed effects of prorenin on vascular resistance in the fetal circulation. All prorenin-related effects appeared to be independent of aldosterone, since, if anything, aldosterone (corrected for renin) induced the opposite.

Hemodynamic maladaptation will affect placenta function and development. Yet, CL number did not relate with either PV or uPVV during the first trimester, which is in agreement with earlier findings, although the measurement technique differed [41]. The fact that prorenin concentrations did correlate negatively with uPVV may be due to its analysis as a continuous variable. Furthermore, it is not unlikely that the consequences of diminished blood flow become fully apparent only in the second and third trimester, since at around 10–12 weeks of gestation the trophoblastic plugs in the maternal spiral arteries dissolve and subsequently the placental blood flow becomes fully established [7]. Here local effects of prorenin in the placenta might be considered as well, given that trophoblast cells are rich in Ang II type 1 receptors [42]. Indeed, Ang II upregulates the production of soluble fms-like tyrosine kinase-1 [43], which through binding of placental growth factor induces an angiogenic disbalance, thus decreasing endothelial cell migration and angiogenesis. Moreover, high placental renin mRNA levels have been reported to associate negatively with trophoblast volume and placental weight [44]. Unfortunately, current technology does not allow proper imaging of placental growth and vascularization during the second and third trimester. Nevertheless, the negative association between first-trimester prorenin concentrations and placental weight in the present study suggest that this line of events continues to be important throughout pregnancy.

Aldosterone-mediated plasma volume expansion is a well-known determinant of the course of pregnancy [45–47]. The positive association between aldosterone (corrected for renin, i.e., independently of Ang II) and PV confirms this view. Here it is important to note that mineralocorticoid receptor blockade is known to decrease umbilical flow [48], while aldosterone additionally induces proliferation of villous trophoblast cells [48].

To the best of our knowledge, our study is the largest that relates the concentrations of RAAS components in early pregnancy to placental development, quantified with well-established non-invasive techniques [4]. Levels fully concur with those reported by others [49]. Results were obtained from a single-center hospital based study, which reduces heterogeneity but also generalizability. Although we were able to correct for multiple confounders, we only focused on one hormonal system, the RAAS. Yet, it is likely that many more contribute to placental development.

In conclusion, excessive RAAS activation, due to the occurrence of > 1 CL, may prevent normal placental development by increasing UtA-RI and PI, although simultaneously aldosterone upregulation counteracts this phenomenon. Since this will ultimately result in SGA, future studies should now evaluate whether other CL-derived factors are involved in this process as well, and to what degree either blocking these factors or reducing the degree of ovarian stimulation (resulting in a lower number of CL) would be of added value to prevent SGA.

## Supplementary Information

**Additional file 1: Supplemental Table 1.** Associations between first-trimester maternal total renin concentrations and trajectories of utero-placental (vascular) development and placental weight. **Supplemental Table 2.** Associations between corpus luteum number at conception and trajectories of utero-placental (vascular) development with additional adjustment for maternal BMI. **Supplemental Fig. 1.** Utero-Placental (vascular) volume trajectory for pregnancies with higher (mean + SD) and lower (mean – SD) total renin (renin + prorenin) concentrations at baseline for 11 weeks gestation in the total study population. In the fully adjusted model 2, high maternal total renin concentrations (mean + 2SD) corresponds to a lower utero-placental vascular volume (at 11 weeks gestation  -3.70 cm^3^) compared to the mean total renin concentrations. Full adjustment includes gestational age, corpus luteum number, maternal age, parity, BMI, smoking, fetal gender and PCOS. **Supplemental Fig. 2.** Correlations by Spearman Rank (*R*) test between maternal plasma total renin (renin+prorenin) concentrations at 11 weeks gestation and placental weight at birth (*n* = 82) by different number of corpora lutea.

## Data Availability

The datasets used and/or analysed during the current study are available from the corresponding author on reasonable request.

## Abbreviations

3D: three-dimensional
Ang: Angiotensin
BMI: Body mass index
CL: Corpus luteum
EDTA: Ethylenediaminetetraacetic acid
ET: Embryo transfer
FET: Frozen embryo transfer
FGR: Fetal growth restriction
FSH: Follicle-stimulating hormone
GA: Gestational age
GnRH: Gonadotropin-releasing hormone
hCG: Human chorionic gonadotropin
hMG: Human menopausal gonadotropin
ICSI: Intracytoplasmic sperm injection
IQR: Interquartile range
IVF: In-vitro fertilization
LH: Luteinizing hormone
LMP: Last menstrual period
MAP: Mean arterial pressure
PE: Preeclampsia
PI: Pulsatility index
PIH: Pregnancy induced hypertension
PTB: Preterm birth
PV: Placental volume
RAAS: Renin-angiotensin-aldosterone system
RI: Resistance index
SD: Standard deviation
SGA: Small-for-gestational age
UmbA: Umbilical artery
uPVV: Utero-placental vascular volume
UtA: Uterine artery
VOCAL: Virtual Organ Computer-aided AnaLysis
VR: Virtual reality
