# Supplementary material for: Corpus luteum number and the maternal renin-angiotensin-aldosterone system as determinants of utero-placental (vascular) development: the Rotterdam Periconceptional Cohort
Source: Reprod Biol Endocrinol. 2021 Nov 4;19:164. doi: 10.1186/s12958-021-00843-9 (PMC8567673; doi:10.1186/s12958-021-00843-9)
Supplement: Supplementary file 1 — Additional file 1: Supplemental Table 1. Associations between first-trimester maternal total renin concentrations and trajectories of utero-placental (vascular) development and placental weight. Supplemental Table 2. Associations between corpus luteum number at conception and trajectories of utero-placental (vascular) development with additional adjustment for maternal BMI. Supplemental Fig. 1. Utero-Placental (vascular) volume trajectory for pregnancies with higher (mean + SD) and lower (mean – SD) total renin (renin + prorenin) concentrations at baseline for 11 weeks gestation in the total study population. In the fully adjusted model 2, high maternal total renin concentrations (mean + 2SD) corresponds to a lower utero-placental vascular volume (at 11 weeks gestation -3.70 cm3) compared to the mean total renin concentrations. Full adjustment includes gestational age, corpus luteum number, maternal age, parity, BMI, smoking, fetal gender and PCOS. Supplemental Fig. 2. Correlations by Spearman Rank (R) test between maternal plasma total renin (renin+prorenin) concentrations at 11 weeks gestation and placental weight at birth (n = 82) by different number of corpora lutea. [file 12958_2021_843_MOESM1_ESM.docx]

**SUPPLEMENTAL MATERIAL**

**Corpus luteum number and the maternal renin-angiotensin-aldosterone system as determinants of utero-placental (vascular) development: the Rotterdam Periconceptional Cohort**

Running title: First-trimester maternal RAAS activation and utero-placental (vascular) development.

Rosalieke E. Wiegel^1^, Maud J.H. Karsten^1^, Igna F. Reijnders^1^, Lenie van Rossem^1^, Sten P. Willemsen^1,2^, Annemarie G.M.G.J. Mulders^1^, Anton H.J. Koning^3^, Eric A.P. Steegers^1^, A.H. Jan Danser^4*^, Régine P.M. Steegers-Theunissen^1*^

* These authors contributed equally.

Author affiliations:

^1^Department of Obstetrics and Gynecology, Erasmus MC, University Medical Center, Rotterdam, The Netherlands

^2^Department of Biostatistics, Erasmus MC, University Medical Center, Rotterdam, The Netherlands

^3^Department of Pathology, Clinical Bioinformatics Unit, Erasmus MC, University Medical Center, Rotterdam, the Netherlands ^4^Department of Internal Medicine, Erasmus MC, University Medical Center, Rotterdam, the Netherlands

Correspondence:

Professor Régine P.M. Steegers-Theunissen, MD, PhD. Department of Obstetrics and Gynecology, Erasmus MC, University Medical Center, Rotterdam, The Netherlands.

Postal address: Dr. Molewaterplein 40, 3015 GD Rotterdam, The Netherlands.

Mobile: +31 6 12472643 Email: r.steegers@erasmusmc.nl

**SUPPLEMENTAL MATERIAL**

**Supplemental table 1.** Associations between first-trimester maternal total renin concentrations and trajectories of utero-placental (vascular) development and placental weight.

|  | | **Model 1** | | **Model 2** | |
| --- | --- | --- | --- | --- | --- |
|  |  | Beta (95% CI) | *p*-value | Beta (95% CI) | *p*-value |
| **First-trimester trajectories** (*n*=151) | | | | | |
| **PV (∛cm^3^)** | Log [Total renin (pg/mL)] | -0.04 (-0.20; 0.12) | 0.62 | -0.14 (-0.34; 0.06) | 0.17 |
| **uPVV (∛cm^3^)** | Log [Total renin (pg/mL)] | -0.09 (-0.28; 0.11) | 0.37 | -0.22 (-0.44; -0.002) | **0.04*** |
| **Total pregnancy trajectories** (*n*=201) | | | | | |
| **Log [UtA-RI]** | Log [Total renin (pg/mL)] | 0.04 (0.002; 0.074) | **0.04*** | 0.04 (0.001, 0.08) | **0.04*** |
| **Log [UtA- PI]** | Log [Total renin (pg/mL)] | 0.05 (-0.02, 0.13) | 0.18 | 0.08 (0.01, 0.17) | **0.04*** |
| **Log [MAP (mmHg)]** | Log [Total renin (pg/mL)] | -0.03 (-0.06, -0.01) | **0.003*** | -0.02 (-0.04, 0.01) | 0.15 |
| **Second-and third trimester trajectories** (*n*=201) | | | | | |
| **Log [UmbA-RI]** | Log [Total renin (pg/mL)] | 0.01 (-0.004, 0.03) | 0.15 | 0.02 (-0.001, 0.04) | 0.06 |
| **Log [UmbA-PI]** | Log [Total renin (pg/mL)] | 0.04 (-0.003, 0.09) | 0.07 | 0.05 (0.001, 0.11) | **0.04*** |
| **At Birth** (*n*=82) | | | | | |
| **Placental weight (gram)** | Log [Total renin (pg/mL)] | -58.25 (-120.39, 3.89) | 0.07 | -97.33 (-166.59; -28.07) | **0.006*** |

Table shows the effect estimates of the repeated measurements model for the associations of logarithmic transformed maternal total renin (renin + prorenin) concentrations at 11 weeks gestation with trajectories of PV and uPVV during the first-trimester, UtA RI/PI and MAP throughout pregnancy and UmbA RI/PI during the second-and third trimester in the total study population and the linear model with placental weight at birth. Model 1: adjusted for gestational age, Model 2: adjusted model for gestational age, corpus luteum number, maternal age, maternal smoking, BMI, parity, fetal gender and polycystic ovary syndrome. **p* < 0.05. CI = confidence interval; PV, placental volume; uPVV, utero-placental vascular volume; UtA, uterine artery; RI, restistance index; PI, pulsatility index; MAP, mean arterial pressure; UmbA = umbilical artery

**Supplemental table 2.** Associations between corpus luteum number at conception and trajectories of utero-placental (vascular) development with additional adjustment for maternal BMI.

|  | | Model 1 | | | Model 2 | |
| --- | --- | --- | --- | --- | --- | --- |
|  |  | Beta (95% CI) | *p*-value | | Beta (95% CI) | *p*-value |
| Total pregnancy trajectories (n=201) | | | | | | |
| Log [UtA-RI] | 1 CL (*n*=142) | *Reference* | | *Reference* | | |
|  | 0 CL (*n*=8) | **-0.13** **(-0.21, -0.05)** | **0.002*** | | **-0.12 (-0.20, -0.04)** | **0.004*** |
|  | >1 CL (*n*=51) | -0.004 (-0.04, 0.03) | 0.83 | | 0.01 (-0.03, 0.04) | 0.97 |
| Log [UtA-PI] | 1 CL (*n*=142) | *Reference* | | *Reference* | | |
|  | 0 CL (*n*=8) | **-0.21** **(-0.39, -0.04)** | **0.02*** | | **-0.18** **(-0.36, -0.01)** | **0.04*** |
|  | >1 CL (*n*=51) | -0.03 (-0.11, 0.05) | 0.42 | | -0.02 (-0.10, 0.06) | 0.60 |
| Log [MAP] | 1 CL (*n*=142) | *Reference* | | *Reference* | | |
|  | 0 CL (*n*=8) | -0.01 (-0.06, 0.04) | 0.73 | | 0.01 (-0.04, 0.06) | 0.62 |
|  | >1 CL (*n*=51) | -0.02 (-0.04, 0.01) | 0.13 | | -0.01 (-0.03, 0.01) | 0.46 |

Table shows the effect estimates of the repeated measurements model for the associations of corpus luteum number at conception and trajectories of UtA RI/PI and MAP throughout pregnancy in the total study population. Model 1: adjusted for gestational age, Model 2: adjusted model for gestational age and maternal BMI. **p* <0.05. CI = confidence interval; UtA, uterine artery; RI, restistance index; PI, pulsatility index; MAP, mean arterial pressure.


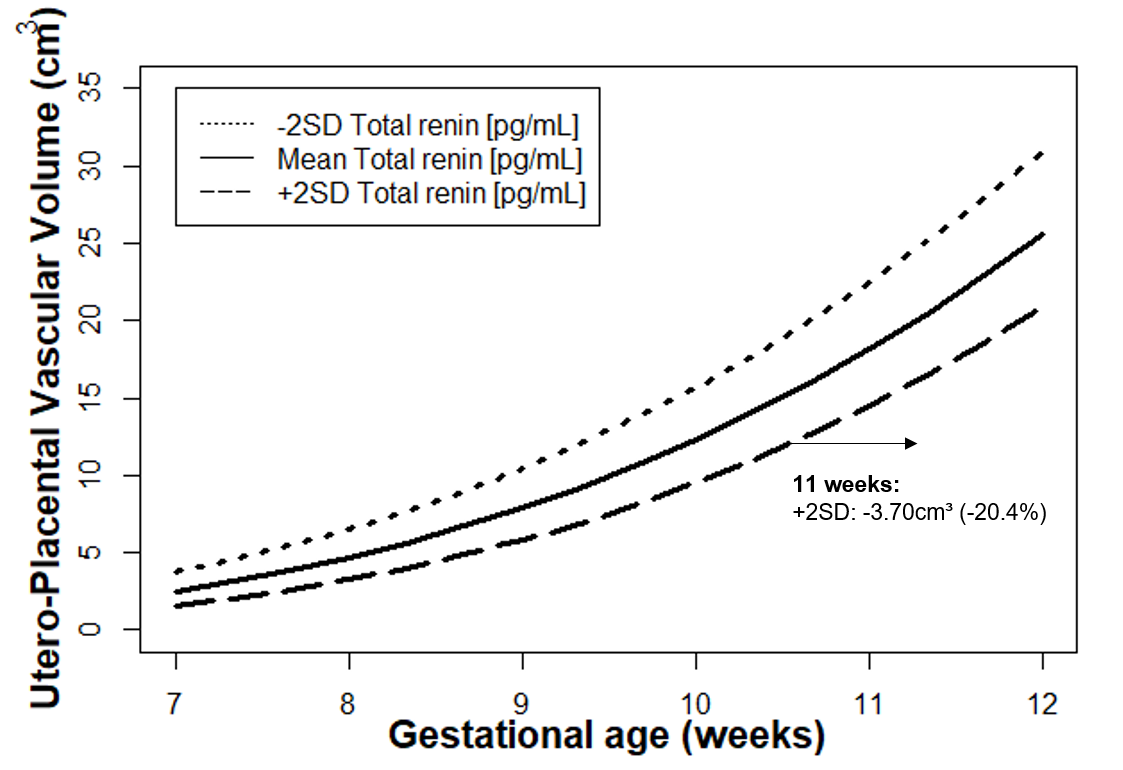


**Supplemental Figure 1.** Utero-Placental (vascular) volume trajectory for pregnancies with higher (mean +SD) and lower (mean – SD) total renin (renin + prorenin) concentrations at baseline for 11 weeks gestation in the total study population. In the fully adjusted model 2, high maternal total renin concentrations (mean+2SD) corresponds to a lower utero-placental vascular volume (at 11 weeks gestation -3.70cm³) compared to the mean total renin concentrations. Full adjustment includes gestational age, corpus luteum number, maternal age, parity, BMI, smoking, fetal gender and PCOS.


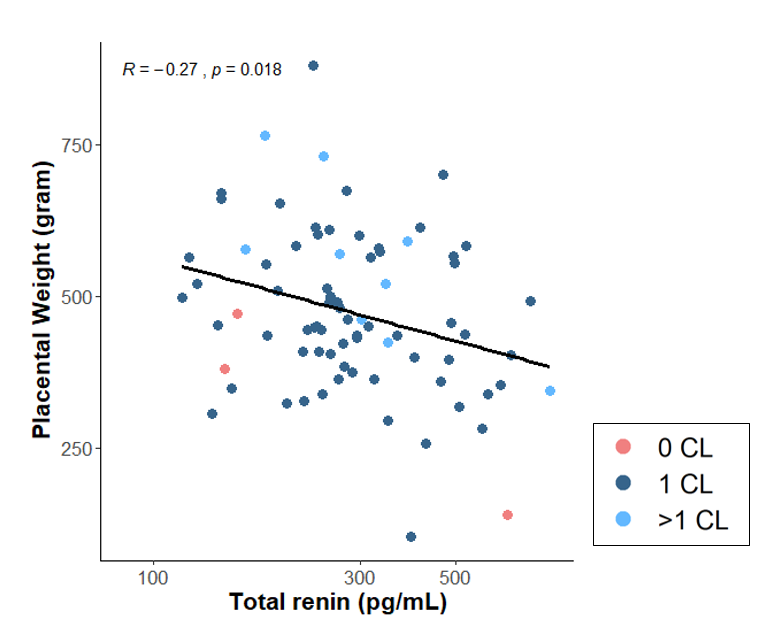


**Supplemental Figure 2.** Correlations by Spearman Rank (*R*) test between maternal plasma total renin (renin+prorenin) concentrations at 11 weeks gestation and placental weight at birth (*n*=82) by different number of corpora lutea.
